# How armed conflict shapes women’s family planning and abortion uptake and decision-making: a scoping review

**DOI:** 10.1080/16549716.2026.2726694

**Published:** 2026-09-04

**Authors:** Argyro Pavlidou, Jenny Hedström, Tobias Herder

**Affiliations:** a Social Medicine and Global Health, Department of Clinical Sciences, Malmö, Faculty of Medicine, Lund University, Malmö, Sweden; b IHE – The Swedish Institute for Health Economics, Lund, Sweden; c Department of War Studies, Swedish Defence University, Stockholm, Sweden

**Keywords:** Reproductive health, SRHR, contraceptives, armed conflict, war

## Abstract

Armed conflict profoundly affects women’s sexual and reproductive health and rights, yet the pathways through which conflict shapes family planning and abortion uptake and decision-making remain insufficiently understood. This scoping review aimed to identify and map the available evidence on how armed conflict influences women’s use of, access to, and decisions about family planning and abortion in conflict-affected settings. Following JBI guidance and -ScR reporting standards, searches were conducted in Web of Science, Scopus, PubMed and Embase for peer-reviewed articles published in English between 2014 and 2024. Studies were eligible if they included primary data on women living in conflict-affected settings or internally displaced populations, treated conflict as an exposure or shaping condition, and addressed family planning, contraception, sterilisation, abortion or post-abortion care. Thirty studies were included, and the data were synthesised using a thematic synthesis approach. Relevant findings from each article were organised into subcategories based on their commonalities under two main themes: a) Factors shaping the uptake of and decision-making about family planning, including contraception and sterilisation, and b) Factors shaping the uptake of and decision-making about abortion. The results highlight that armed conflict does not automatically affect reproductive health in a single direction; rather, its effects depend on how violence, service availability, gendered power relations, social norms and social conditions interact. This study calls for further research to better understand the effects of conflict on women’s reproductive health and autonomy, and inform tailored humanitarian interventions to address these needs.

## Paper Context

- **Main findings:** Armed conflict shapes women’s uptake of and decision-making about family planning and abortion in complex and context-specific ways. Its effects depend on how health services, violence, poverty, social norms, gender relations and humanitarian responses interact in particular settings.
- **Added knowledge:** This scoping review maps evidence on family planning and abortion in conflict-affected settings and shows that conflict does not influence reproductive health in one uniform direction. The review highlights how conflict can both restrict access to care and reshape the conditions under which women make reproductive decisions.
- **Global health impact for policy and action:** Policies and programmes in conflict-affected settings should address not only service availability, but also insecurity, poverty, gender-based violence, social norms and women’s reproductive autonomy. Responses should be adapted to local conditions and ensure accessible, acceptable and rights-based family planning and abortion-related care.

## Background

Sexual and reproductive health and rights (SRHR) are an integral part of achieving good health and well-being, and is widely held to contribute to sustainable development and gender equality [1]. As defined in the ICPD Programme of Action, reproductive rights *‘rest on the recognition of the basic right of all couples and individuals to decide freely and responsibly the number, spacing and timing of their children and to have the information and means to do so, and the right to attain the highest standard of sexual and reproductive health. It also includes their right to make decisions concerning reproduction free of discrimination, coercion and violence, as expressed in human rights documents’* [2]. Access to family planning services, such as contraceptive methods, and reduced fertility, e.g. delay and/or decline in childbearing, can have a positive effect on women’s equality and health, as well as their children’s health and education, with potential benefits for society and economy [3].

In conflict-affected contexts, however, women’s ability to realise SRHR is often severely constrained [4]. Armed conflict may disrupt health systems, damage infrastructure, limit the availability of trained healthcare providers and reduce access to essential maternal and reproductive health services [5]. At the same time, conflict may increase exposure to gender-based and sexual violence, transactional sex, unintended pregnancies, unmet family planning needs and abortion-related risks such as severe bleeding and infection [6–8]. These conditions create reduced agency over reproductive decisions which may contribute to higher mortality and poverty rates, while adding to women’s exclusion from decision-making on issues regarding peace and conflict [4].

Different types of conflict and involved armed groups may use different tactics and policies when it comes to women’s SRHR and specifically to reproductive health access and uptake [4]. Examples of these may include attacks on clinics, looting, abduction and killing of healthcare providers or, in conflicts motivated by ethnic identity, favouritism and discrimination in providing services to women based on their ethnic origin [5]. Furthermore, women’s reproductive rights and autonomy can be at stake due to pro-natalist notions urging them to produce more soldiers to support the armed conflict or in order to repopulate the nation and strengthen the ethnic population, carrying over into post-conflict periods [9].

However, even within the same country, conflict-affected areas may receive disproportionately higher levels of humanitarian assistance and investment than regions located farther from the conflict. While it is evident that conflict profoundly shapes women’s access to SRHR, we do not know enough about how, or why, war affects their access to family planning and abortion services. In this scoping review we therefore seek to identify and map the type and scope of the evidence regarding family planning and abortion in conflict-affected settings, by asking: How does conflict shape the uptake of and decision-making about family planning and abortion among women in conflict-affected contexts?

## Methods

A scoping review design was chosen because it is well suited to identifying, mapping and summarising the type and scope of available evidence in an emerging and heterogeneous field. Given that the aim of this review was not to assess intervention effectiveness or produce pooled estimates, but rather to clarify how armed conflict has been studied in relation to women’s family planning and abortion uptake and decision-making, a scoping review approach was considered appropriate. The review was conducted following the JBI Manual for Evidence Synthesis [10] and reported according to the Preferred Reporting Items for Systematic reviews and Meta-Analyses extension for Scoping Reviews (PRISMA-ScR) guidelines [11].

### Search strategy

**Figure 1.**
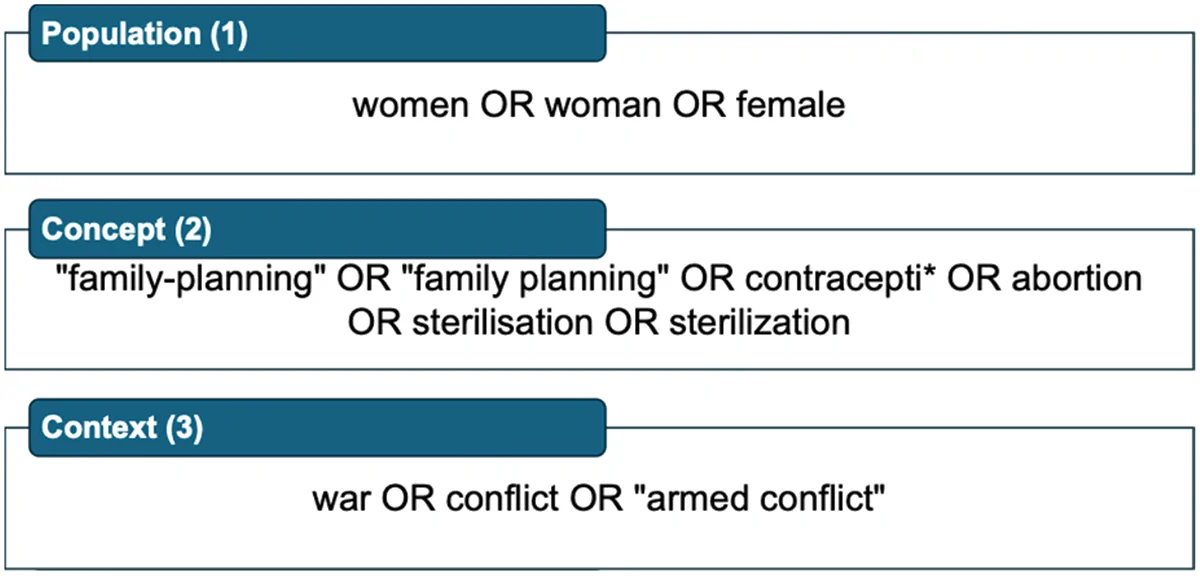
Keywords of the search strategy divided into 3 blocks. *The 3 blocks were connected with the word “AND” in the search, i.e. [1] AND [2] AND [3].*

The search string was developed using relevant keywords that were searched in title and abstract. The keywords were divided into 3 search blocks related to our key concepts: 1) being a woman (population), 2) reproductive concept (concept) and 3) conflict (context). The reproductive health concepts of interest included: family planning, contraception, sterilisation and abortion, as shown in Figure 1.

Four electronic databases were searched for eligible articles: Web of Science, Scopus, PubMed and Embase. The search was limited to peer‑reviewed articles published from 2014 to 2024 and written in English. This time period was chosen to capture contemporary evidence on armed conflict and reproductive health, including studies published during a period of increasing attention to women’s sexual and reproductive health and rights in conflict-affected settings. The search strategy was developed and refined in June 2024 and executed on 3 July 2024.

### Screening and eligibility criteria

Articles identified through the database searches were first exported into EndNote and then organised in four different groups based on the database from which they were retrieved. The records were then imported into Covidence, where duplicates were removed and both title and abstract screening, as well as the full-text reviews were conducted. Initial title and abstract screening were conducted by AP. Full‑text screening was then conducted by AP and TH separately. Any disagreements during screening were discussed with JH until agreement was reached. Data extraction and evidence synthesis were initially conducted by AP and subsequently reviewed by TH.

Articles were eligible for inclusion in the analysis if they met the following criteria: 1) they included women of any age residing in a conflict-affected setting or who were Internally Displaced Persons (IDPs); 2) conflict was discussed or treated as an exposure, i.e. as a factor shaping the uptake and decision-making over family planning and abortion; 3) the context was explicitly described as conflict-affected; 4) the study included primary data on uptake or decision-making, i.e. use of services, intentions and/or attitudes over family planning and fertility, contraception, sterilisation or abortion; 5) data were derived from women themselves or from registries containing data on women’s reproductive health outcomes; and 6) the article was written in English. The choice of only including women in conflict-affected settings or IDPs was made in order to identify evidence that are specific to these settings’ challenges, as opposed to the, potentially more complex, challenges faced by women who are migrants or refugees, such as challenges throughout their journey or challenges related to being in a foreign country, which are related to different dynamics. In addition, if women had fled to more wealthy countries, the challenges or provision of SRHR services may have been differently affected than being in less affluent, conflict-affected settings with disrupted health systems. Therefore, we decided to focus on the challenges that are faced by women while they are still residing in the conflict-affected countries, where resources are most likely less, and SRHR needs might be more easily neglected.

Studies were excluded if they: 1) focused primarily on refugees or migrants who moved to other countries outside of the conflict-affected context; 2) did not discuss how exposure to conflict affected the outcomes; 3) were set in other humanitarian settings or did not mention that the setting was conflict-affected; 4) did not include original data on the outcomes of interest; 5) did not include primary data or 6) were not written in English. Reviews, commentaries, editorials and opinion pieces were therefore excluded.

Both qualitative and quantitative articles were included, provided that the eligibility criteria were fulfilled. As common in scoping reviews, no formal critical appraisal was carried out [10]. To reduce the risk of including studies published in potentially predatory journals, journals were checked against the Norwegian Register for Scientific Journals, Series and Publishers. The register classifies scholarly publication channels according to defined quality criteria, with Level 1 indicating recognised scholarly publication channels and Level 2 indicating channels with particularly high scholarly standing. Only journals classified at Level 1 or 2 at the time of publication were eligible for inclusion [12,13].

### Data extraction and synthesis

Data were extracted into a charting table developed for this review (Table 1). The extracted data included country of study, population and sample size, study methodology, reproductive health concept studied, summary of the conflict-related findings, information on ethical approval and the ranking of the journal in which the study was published according to the Norwegian Register for Scientific Journals, Series and Publishers.

**Table 1.** Characteristics of included studies on family planning and abortion in conflict-affected settings.

#	Citation	Title	Country	Population & sample size	Method	Reproductive health concept	Summary of conflict-related findings	Norwegian ranking^1^
1	Akseer et al. [19]	Association of Exposure to Civil Conflict With Maternal Resilience and Maternal and Child Health and Health System Performance in Afghanistan	Afghanistan	Women aged 12–49 years, *N* = 64 815	Quantitative, population-based, sequential cross-sectional	Contraceptive use	Contraceptive use improved overall between 2003 and 2018. Provinces experiencing severe- to moderate-intensity conflict showed slower progress when compared to provinces with minimal-intensity conflict.	2019:1 2024:1
2	Akseer et al. [22]	Women, children and adolescents in conflict countries: an assessment of inequalities in intervention coverage and survival	All LMIC (2019 ranking)	Mothers aged 15–49, *N* ≈ 3 800 000	Quantitative, ecological	Family planning	Inequalities disadvantaging the poorest (vs richest), least educated (vs most educated) and rural mothers (vs urban) were significantly higher in conflict countries compared to non-conflict countries for satisfied family planning needs.	2020:1 2024:1
3	Asefa et al. [39]	The magnitude of gender-based violence, health consequences, and associated factors among women living in post-war woredas of North Shewa zone, Amhara, Ethiopia, 2022	Ethiopia	Women aged 16–80, *N* = 845	Quantitative, community-based, cross-sectional	Abortion	The study found increased prevalence of gender-based violence (GBV) during the conflict with abortion being one of the common consequences of experiencing GBV.	2024:1
4	Bassel [32]	Acts of Truth Telling and Testimony in the Conceptualisation of Reparations in Post-conflict Peru	Peru	Indigenous women who underwent forced sterilization in the 1990:s.	Qualitative analysis of testimonials	Sterilisation	Forced sterilisation tactics were used against indigenous women, mainly Quechua speaking, to suppress the rural regions and indigenous communities. Indigenous, predominantly illiterate, women were targeted, lied to, bribed and threatened in order to force them into undergoing sterilisation surgeries.	2020:1 2024:1
5	Boddam-Whetham et al. [20]	Vouchers in Fragile States: Reducing Barriers to Long-Acting Reversible Contraception in Yemen and Pakistan	Yemen, Pakistan	Women from Yemen and Pakistan who participated in voucher programs aimed at reducing barriers LARC and permanent methods (PMs) of family planning. In Yemen 56 000 vouchers were distributed, in Pakistan 83 920 vouchers.	Evaluation / Quasi-experimental study.	Family planning	Voucher programs can keep vital funds flowing to public health care providers in times of conflict and give women alternatives in places where government facilities are temporarily closed due to conflict. The authors encourage program implementers in fragile settings to consider vouchers to reduce barriers and improve access to family planning services.	2016:1 2024:1
6	Bove et al. [30]	Mothers at peace: International peacebuilding and post-conflict fertility	Liberia	Women aged 15–45, *N* = 17 226	Quantitative, sequential DHS survey data combined with geocoded data on deployment of UN peacekeepers.	Contraceptive use	Exposure to peacekeeping was linked to improved health outcomes for both mothers and children, as indicated by improved indicators and increased contraceptive use, while conflict has negative effects on contraceptive use.	2024:1
7	Burkhardt et al. [37]	Sexual violence-related pregnancies in eastern Democratic Republic of Congo: a qualitative analysis of access to pregnancy termination services	Democratic Republic of Congo	38 women raising children conceived from sexual violence-related pregnancies (SVRPs) and 17 women who had terminated SVRPs.	Qualitative, thematic content analysis of semi-structured interviews	Abortion	Majority of women who had terminated SVRPs had used traditional herbs, and evidence-based methods were not mentioned. Some women had attempted to terminate unsuccessfully, and some continued their pregnancies due to fear of death from unsafe abortion methods. Attitudes towards abortion were considered different when the pregnancy resulted from sexual violence.	2016:1 2024:1
8	Casey et al. [27]	Twelve-month contraceptive continuation among women initiating short- and long-acting reversible contraceptives in North Kivu, Democratic Republic of the Congo	Democratic Republic of Congo	Women who began using short-acting contraceptive methods (pills, injectables) 12–18 months prior, *N* = 304. Women who began using long-acting reversible contraceptive methods (intra-uterine devices, implants) 12–18 month prior, *N* = 244.	Quantitative, retrospective.	Contraceptive use	Long-acting reversible contraceptive acceptors were more likely to have been displaced in the last year. Continuation of contraception use was similarly high across displacement status.	2017:1 2024:1
9	Casey et al. [28]	Meeting the demand of women affected by ongoing crisis: Increasing contraceptive prevalence in North and South Kivu, Democratic Republic of the Congo	Democratic Republic of Congo	Women aged 15–49 from six health zones in North and South Kivu, *N* = 3271	Quantitative, cross-sectional population-based survey	Contraceptive use	Current modern contraceptive use and LAPM use were high. LARC use was high across all age groups. These results were accomplished across all six health zones despite their varied sociodemographic characteristics and different experiences of conflict and displacement. Use of modern contraception and of LAPM was comparatively high in the health zones most affected by the conflict and displacement.	2019:1 2024:1
10	Casey et al. [25]	Contraceptive use among adolescent and young women in North and South Kivu, Democratic Republic of the Congo: A cross-sectional population-based survey	Democratic Republic of Congo	Women aged 15–24 years from six health zones in North and South Kivu, *N* = 1022	Quantitative, cross-sectional population-based survey	Contraceptive use	Displacement was not associated with modern contraceptive use.	2020:2 2024:2
11	Casey et al. [44]	Community perceptions of the impact of war on unintended pregnancy and induced abortion in Protection of Civilian sites in Juba, South Sudan	South Sudan	Women aged 18–45 from *Protection of Civilan* sites, *N* = 18	Qualitative, thematic content analysis of focus group discussions	Abortion	Unintended pregnancy and induced abortion were considered to have increased during the current conflict and due to the changes caused by war. Less condemnatory views were expressed regarding the reasons why women have abortions during current conflict.	2022:1 2024:1
12	Casey et al. [43]	‘You must first save her life’: community perceptions towards induced abortion and post-abortion care in North and South Kivu, Democratic Republic of the Congo	Democratic Republic of Congo	Women aged 18–24 (*n* = 61) and aged 25–45 (*n* = 63) from six health zones in North and South Kivu	Qualitative, thematic content analysis of focus group discussions	Abortion and post-abortion care	More lenient attitudes towards induced abortion were present when the reason for unintended pregnancy was rape by armed forces.	2019: 1 2024: 1
13	Curry et al. [21]	Delivering high-quality family planning services in crisis-affected settings II: results	Democratic Republic of Congo, Mali, Chad, Djibuti, Pakistan	Women age 15–49 who were new users of modern contraceptive methods at project sites (*N* = 52616)	Quantitative analysis of client registry data	Contraceptive use	Decreases in modern contraceptive uptake were experienced at the program implementation sites in Democratic Republic of Congo due to changes in active conflict.	2015:1 2024:1
14	Elmusharaf et al. [29]	Social and traditional practices and their implications for family planning: a participatory ethnographic study in Renk, South Sudan	South Sudan	Women attending a participatory training workshop (*n* = 14) each interviewing three of their friends (*n* = 42)	Qualitative, Participatory Ethnographic Evaluation and Research (PEER), with data analyzed though thematic analysis.	Family planning	As a result of wars, people are under pressure to increase their family sizes, to compensate for lives lost and high child mortality.	2017:1 2024:1
15	Erickson et al. [26]	Structural determinants of dual contraceptive use among female sex workers in Gulu, northern Uganda	Uganda	Female sex workers (*N* = 400)	Quantitative, cross-sectional survey data	Contraceptive use	Abduction by the Lord’s Resistance Army and having lived in an IDP camp were not associated with dual contraceptive use.	2015:1 2024:1
16	Erickson et al. [36]	Incarceration and exposure to internally displaced persons camps associated with reproductive rights abuses among sex workers in northern Uganda	Uganda	Female sex workers (*N* = 400)	Quantitative, cross-sectional survey data	Abortion	Historical exposure to living in an IDP was associated with increased odds of unsafe abortion.	2017:1 2024:0
17	Kidman et al. [24]	Intimate partner violence, modern contraceptive use and conflict in the Democratic Republic of the Congo	Democratic Republic of Congo	Ever married women age 15–49 (*N* = 2859)	Quantitative, DHS and ACLED data.	Contraceptive use	Modern contraceptive use was slightly higher in conflict clusters as compared to non-conflict clusters (8% versus 4%). In South Kivu and North Kivu, which are engaged in widespread conflict the highest rates of current modern contraceptive use (15–16%) were measured.	2015: 2 2024: 2
18	Koya & Mpinga [40]	Perceptions of the rape crisis in the Eastern Democratic Republic of Congo: A community-based approach using an opportunistic design	Democratic Republic of Congo	Girls and women in the Orientale, North and South Kivu provinces (*N* = 1495)	Quantitative, cross-sectional community survey	Abortion	Abortion as one of the main consequences of rape, perpetrated mainly by members of army/security forces/militias and followed by civilians, was reported at 10.37%.	2022:1 2024:1
19	Namasivayam et. al. [15]	The Effect of Armed Conflict on the Utilization of Maternal Health Services in Uganda: A Population-based Study	Uganda	Women aged 15–49 (*N* = 36251)	Quantitative, DHS survey data	Contraceptive use	Odds of using contraception in the north of Uganda were significantly lower compared to the rest of the country Odds for fertility-related concerns, opposition to use and lack of knowledge were significantly lower among women in the North across all time points in both analyses, and in 2006 lack of access was significantly lower for women in the North as well.	2017: 1 2024: 1
20	O’Brien [35]	The Consequences of the Tajikistani Civil War for Abortion and Miscarriage	Tajikistan	Women aged 15–49 who have ever been pregnant and reached menarche during or after the Tajikistani civil war. (*N* = 1445)	Quantitative, Retrospective survey data and Uppsala Conflict Data Program’	Abortion	Direct conflict event exposure in a woman’s district was not significantly associated with a higher likelihood to induce abortion, but the spillover effects in the neighbouring districts were significant. When neighbouring districts experienced more conflict events, the spillover effects include an increase in abortion. These results did not hold for women who reached menarche after the civil war ended.	2021: 1 2024: 1
21	Oladeji et al. [42]	Sexual Violence-Related Pregnancy Among Internally Displaced Women in an Internally Displaced Persons Camp in Northeast Nigeria	Nigeria	Women with self-reported SVRP at primary health clinic in the dalori IDP camp (*N* = 47)	Quantitative, retrospective chart review	Abortion	All women were abducted from their communities and held hostage for more than 2 years before being liberated by the military. It was documented that all participants requested for termination of pregnancy at the initial contact stating that they were not willing to have the babies due to the circumstances associated with them becoming pregnant.	2021: 1 2024: 2
22	Ononokpono et al. [18]	Non-use of modern contraceptives among women in humanitarian contexts: evidence from a qualitative study in Akwa Ibom State, Nigeria	Nigeria	Women aged 20–49 (40)	Qualitative, Thematic analysis of focus group discussions	Contraceptive use	Reporting of non-use of contraceptives due to the need for more children, as large families are fearful during local conflicts in the community. Some of the participants reported limited access to health facilities and lack of workers to take care of the reproductive health care needs of women. The nearby health centres were seen to be ill-equipped and facilities offering quality service were far.	2023: 1 2024: 1
23	Perera et al. [38]	Barriers to seeking post-abortion care in Paktika Province, Afghanistan: a qualitative study of clients and community members	Afghanistan	Women who had received post-abortion care aged 18 to 35, Married women aged 18–45 (*N* = 50)	Qualitative, Thematic content analysis of individual interviews and focus group discussions.	Post-abortion care	While barriers to accessing and using services are not unique to conflict, and some are not specific to post-abortion care, others, particularly those related to stigma surrounding abortion, may be more prevalent in this context.	2021: 1 2024: 1
24	Rivillas et al. [23]	How do we reach the girls and women who are the hardest to reach? Inequitable opportunities in reproductive and maternal health care services in armed conflict and forced displacement settings in Colombia	Colombia	Women affected by forced displacement and sexual and gender-based violence in conflict settings in Colombia (2005, *N* = 258 714; 2010, *N* = 131 345; 2015, *N* = 96 422))	Quantitative cross-sectional secondary survey data from 2005, 2010 and 2015)	Family planning	In 2005 women affected by forced displacement and GBV in conflict settings had higher unmet family planning needs.	2018: 1 2024: 1
25	Svallfors [34]	Contraceptive choice as risk reduction? The relevance of local violence for women’s uptake of sterilization in Colombia	Colombia	Women aged 13–49 (*N* = 113 403)	Quantitative, DHS survey data and Uppsala Conflict Data Program	Sterilisation	A consistent, positive and statistically significant association found between conflict events and the likelihood of sterilization. The effects were stronger when conflict events were measured over recent periods.	2022: 2 2024: 2
26	Svallfors et al. [33]	Armed conflict, insecurity, and attitudes toward women’s and girls’ reproductive autonomy in Nigeria	Nigeria	Women aged 18 and above (*N* = 534)	Quantitative, Cross-sectional from World Value Survey and Uppsala Conflict Data Program	Contraceptive use, Abortion	Women exposed to the highest levels of conflict were more likely to support safe abortion access Both women who perceived their neighbourhoods as safe and those who perceived them as unsafe, supported safe abortion access when exposed to conflict	2024:2
27	Svallfors and Billingsley [16]	Conflict and Contraception in Colombia	Colombia	Women aged 13–49 (*N* = 103 759)	Quantitative, DHS survey data and Uppsala Conflict Data Program	Contraceptive use	The likelihood of using modern contraceptives was lower in conflict-affected areas. Although modern contraceptive use increased from 1990 to 2016, the progress was hindered by the effect of armed conflict. Fertility desire was found to be a mediator, increasing due to conflict, which could partly explain the reduction in contraceptive use	2019: 1 2024: 1
28	Tenaw et al. [41]	Medical and psychological consequences of rape among survivors during armed conflicts in northeast Ethiopia	Ethiopia	Women being victims of rape (*N* = 271)	Mixed method, Quantitative analysis of health records, and qualitative phenomenological approach to analysing individual interviews.	Abortion	Among the survivors, 10.7% of the women were pregnant, as a consequence of rape by armed group soldiers. Among pregnant women, 44.8% terminated the pregnancy through induced medical abortion	2022: 1 2024: 1
29	Torrisi [17]	Violent instability and modern contraception: Evidence from Mali	Mali	Women aged 15–49 (*N* = 17 570)	Quantitative, DHS survey data and Uppsala Conflict Data Program	Contraceptive use, Family planning	Conflict was negatively associated with modern contraceptive use, especially for short-acting methods. The conflict was linked to a greater risk of unwanted pregnancy and intention to use modern contraceptive methods. Conflict negatively influenced women’s knowledge about where to access modern contraceptives and increased the reporting of ‘supply-side’ barriers as reasons for non-use	2024:2
30	Wirtz et al. [31]	Gender-based violence in conflict and displacement: qualitative findings from displaced women in Colombia	Colombia	Internally displaced women who self-identified as survivors of gender based violence, aged 18–61. (*N* = 35)	Qualitative, Grounded theory of individual interviews	Contraceptive use, abortion	Women faced gender-based violence in both conflict and displacement settings, including forced labour, forced abortion and control over contraceptive use. In several cases, forced sex resulted in unintended or unplanned pregnancy. Women, however, found tactics to overcome their partners’ control of their reproductive decisions, e.g. by choosing contraceptive methods that are not detectable.	2014: 1 2024: 2

^1^The Norwegian Register for Scientific Journals, Series and Publishers classifies scholarly publication channels according to defined quality criteria, with Level 1 indicating recognised scholarly publication channels and Level 2 indicating channels with particularly high scholarly standing. Level 0 indicate that one or more of the quality criteria of the register are not fulfilled or documented and the publication channel is not recognized as an academic publication channel.

We used a thematic synthesis approach to organize and interpret the extracted findings across the included studies [14]. Relevant findings from each article were first summarized and collated in relation to the review aim and research question. The extracted findings were then organised into subcategories based on their commonalities under two main themes: a) Factors shaping the uptake of and decision-making about family planning, including contraception and sterilisation, and b) Factors shaping the uptake of and decision-making about abortion.

## Results

**Figure 2.**
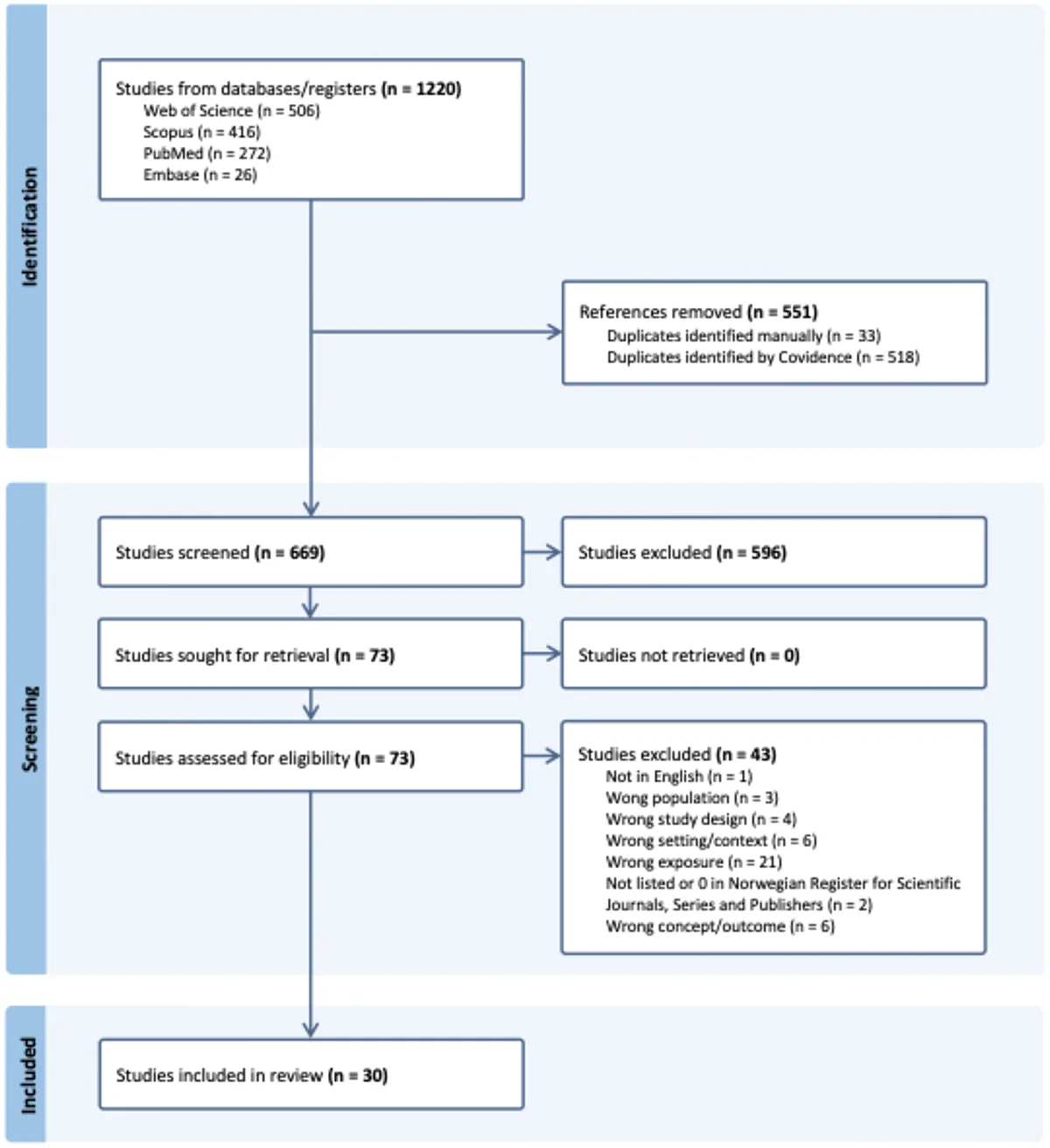
PRISMA chart.

Our literature search retrieved 1220 articles. After deduplication, 669 articles were eligible for title and abstract screening, of which 596 were considered irrelevant. In total, 73 research articles were assessed for eligibility through full-text screening and 43 were excluded for various reasons as illustrated in the chart in Figure 2. Finally, 30 were included in this review and are presented in Table 1. The majority of the included studies used quantitative or mixed methodology (*n* = 22; 73%). In addition, most studies solely focused on the reproductive concept of family planning, including contraception and sterilisation (*n* = 18; 60%). Ten articles (33%) focused solely on abortion, including post-abortion care (PAC) and two articles included both family planning- and abortion-related concepts (6%). The ways in which conflict influences and shapes the uptake of and decision-making about family planning and abortion are presented below for each concept separately.

### Family planning

The theme of family planning includes the concepts of contraception and sterilisation, in terms of both uptake of these services, as well as attitudes towards them and fertility intentions. In total, twenty of the included articles concerned these topics; eighteen solely focused on family planning-related concepts, whereas two articles included both family planning and abortion. Conflict was found to affect family planning uptake and attitudes over it in various ways; negatively, positively or not at all, depending on context, as outlined in the following sub-themes.

#### Structural factors

Conflicts have the potential to affect uptake and decision-making regarding family planning in different directions. On the one hand, conflict exposure has been associated with lower contraceptive use [15–17], due to barriers such as distance to health centres, poor quality of health services and financial constraints [18], as well as lack of supplies and healthcare providers [17,18] and lack of information on where to access contraceptives [17]. Previous progresses made in contraceptive uptake was also found to be hindered due to conflict [16,17,19]. Another way through which armed conflict was found to affect contraception uptake was the negative impact conflict had on aid programmes’ reach due to for example fluctuations in the intensity of conflict [20,21]. Moreover, in conflict-affected countries inequalities in terms of satisfied family planning needs were found to be higher when comparing to countries not affected by conflict, favouring the richer, most educated and urban mothers to greater extent compared to women living in poorer, rural households [22]. Within-country inequalities have also been reported in Colombia, with women affected by conflict and displacement having higher unmet family planning needs [23].

In contrast, there are also findings showing that modern contraceptive use has been higher in conflict-affected regions [24], or unaffected. Studies found no association between displacement and modern contraceptive use among women 15–24 years old in the Democratic Republic of Congo [25] or dual contraceptive use among sex-workers in Uganda [26]. Another study from the Democratic Republic of Congo found that long-acting reversible contraception (LARC) users to be more likely to have been displaced during the previous year, but that 12-month contraceptive continuation was similar regardless of displacement status [27]. Additionally, long-acting or permanent method (LAPM) use was high in different zones of conflict-affected regions, regardless of women’s differences in conflict and displacement experience [28]. Voucher programs have been suggested as an effective strategy to reduce barriers to family planning for women in conflict-affected areas, and have been implemented in Yemen and Pakistan when other facilities were closed and funds were limited [20].

#### Militarisation

Both the presence of armed forces and the active participation of civilians in the conflict and military have been found to affect family planning. The experience of conflict impacts family planning for different reasons, such as men going to war and women staying behind, women becoming widows and women bearing less children due to their husbands not returning from conflict [29]. On the other hand, peacekeeping presence has been found to be associated with increased contraceptive use, as humanitarian interventions are enabled in such settings, facilitating access to infrastructure and family planning services [30].

#### Gender and power dynamics

In contexts with more intense conflicts women’s reproductive autonomy has been found to be impacted by lower ability to negotiate condom use and refuse sex [17]. Gender-based violence (GBV) is prevalent in both conflict and displacement settings and one way through which it manifests has been control over contraceptive use [31]. Despite that, women adapt and find ways to overcome this barrier, for example by opting for covert contraceptive methods [31]. Higher risk of unintended pregnancies has been linked to conflict [17] and in several cases these pregnancies were the result of sexual violence [31]. Another form of reproductive control which has been used in conflict contexts is forced sterilisation. This was reported from indigenous women in Peru, where sterilisation by force and torture was documented in one study [32].

#### Attitudes about family planning and fertility intentions

Conflicts also impact attitudes regarding family planning according to several studies. On the one hand, there is a fertility desire and wanting to compensate for family and community members that have been lost due to conflicts [16,29], as well as the desire to create more members in order to be stronger in future conflicts or have help in the IDP camps [18,29]. On the other hand, conflict has been linked to both higher intention of contraceptive use [17] and lower opposition towards contraceptive use [15]. Moreover, women exposed to conflict and living in an area classified as unsafe were more likely to support access to contraception compared to when living in a safe area [33]. A study on sterilisation in Colombia showed that conflict events were associated with a greater probability for sterilisation uptake and the effect was stronger with more recent conflict. Women exposed to the highest intensity of conflict were more likely to become sterilised compared to unexposed women [34].

### Abortion

The theme of abortion includes the concepts of abortion and post-abortion care in terms of both uptake of these services, as well as attitudes towards them. In total, ten of the included articles solely focused on these concepts, whereas two articles included both family planning and abortion. Conflict was found to affect abortion-related concepts in various ways, such as higher rates of unsafe abortion and more lenient attitudes towards abortion, or it did not affect them in a specific way.

#### Exposure to conflict and displacement

Exposure to conflict and its effects on living conditions has been found to shape abortion uptake and decision-making among women. Although living in a conflict-exposed area was not found to increase abortion rates, it was found that conflict in neighbouring areas did [35]. Moreover, historical exposure to living in IDP camps has been found to be associated with higher rates of unsafe abortion [36]. Decision-making over abortion is explained to be influenced by concerns about complications after an abortion when women were left alone as their husbands were away [37]. The only study that focused solely on post-abortion care did not yield any conflict-specific findings, i.e. the factors that influenced post-abortion care were not explicitly conflict-specific [38].

#### Gender-based violence

Gender-based violence, including sexual violence, was found to increase during conflicts [39]. Perpetrators included armed forces, family members or others, and studies reported cases of sexual violence related pregnancies (SVRP) and found abortion as a common consequence of the conflict‑related sexual violence [39–41].

In one study from Nigeria, all women who were abducted and became pregnant during their abduction, requested termination of pregnancy after their liberation [42]. Even when the intention of abortion was present, it was not always possible due to other factors, such as lack of knowledge, religion, refusal from healthcare providers or the law [37,42]. SVRPs were mainly terminated outside the healthcare system and by following traditional instead of evidence-based methods [37]. Furthermore, forced abortion was an additional GBV manifestation in conflict and displacement settings [31].

#### Attitudes about abortion

The presence of sexual violence in conflict contexts was found to influence attitudes about abortion, making women more lenient towards abortion [37,43]. Abortion was seen as an understandable choice and social consequences were alleviated when sexual violence perpetrated by armed forces was the cause of unwanted pregnancy [43].

Attitudes towards abortion were also shaped by life circumstances in IDP camps. Poverty, instability, transactional sex, separation from family and sexual violence affected women’s choices and abortion was then described as a way to protect oneself from disclosure and distance oneself from the perpetrator which was seen as especially important when he belonged to an opposing ethnic group [44]. In addition, changes in norms and customs around marriage due to conflict and poverty, including men’s inability to pay for dowry and marriage, have been described. This was understood as affecting women’s decisions as well as family pressure towards abortion when marriage was not an option [44].

Women living in conflict-affected areas in Colombia were also found to be more likely to support access to safe abortion, an association increasing with conflict intensity. Those exposed to the highest levels of conflicts were more likely to support abortion access compared to unexposed women. Women living in both perceived as safe and unsafe neighbourhoods were supporting safe abortion access when exposed to conflict [33].

## Discussion

The results of this scoping review show how armed conflicts shapes women’s family planning and abortion uptake and decision-making in both complex and context‑specific ways. Rather than assuming that conflict affects family planning and abortion in one direction, the articles reviewed in this study suggest that conflicts reconfigure gender relations, and the social and institutional conditions under which reproductive decisions are and can be made. Across the included studies, women’s access to, use of and decisions about family planning and abortion were sometimes shaped by health service availability, displacement, poverty, insecurity, exposure to violence, humanitarian assistance and changing gender relations. What this makes clear is that armed conflict profoundly restructures women’s access to family planning and abortion, but what direction this will take depends on context. While previous reviews have highlighted that women’s health is particularly vulnerable to the effects of conflict [45], this review clearly shows how this is also the case for women’s reproductive health.

This scoping review contributes to a growing body of literature on public health in war by examining how armed conflict shapes women’s sexual and reproductive health, and it highlights the importance of how not only infrastructure and availability of services is influenced, but also how social factors, attitudes and gender relations are reshaped during conflict. Conflict may, for example, separate partners, increase widowhood, disrupt livelihoods and reshape marriage practices, all of which may influence whether women seek to avoid pregnancy or have more children [see for example, 29]. Moreover, the studies reviewed suggest that norms and attitudes concerning SRHR also are affected by conflict, for example by showing how acceptability towards abortion was higher when unwanted pregnancies occurred during conflict or due to sexual violence in conflict [see for example, 43].

While conflict has previously been reported to limit access to for example contraceptives, conflict may paradoxically also expand access to medicines and health services through humanitarian interventions in displaced communities and other conflict-affected areas. Thus, while previous research demonstrates that war disrupts access to healthcare [45], increasing exposure to intimate partner violence (IPV) [46] and conflict-related sexual violence [47] as well as sexually transmitted infections [48], this review adds that conflicts also shape the meaning of and acceptability of reproductive choices, including abortion. The findings suggest that armed conflict transforms the most intimate dimensions of everyday life, shaping decisions about childbearing and contraceptive use, as well as broader reproductive patterns.

This scoping review identified evidence from several different countries and related to numerous factors that shape women’s autonomy in terms of their SRHR in conflict. However, it was noted that certain countries were represented more often than others in the gathered evidence, while some geographical regions were not represented at all. Although challenging given the conflict-affected character of such settings, further research should focus on exploring the experiences and challenges that women face in conflict-affected contexts in different parts of the world, in order to inform interventions that are context-specific and tailored to local needs. In addition, it would be valuable to investigate more the differences under certain types of conflicts, such as conflicts with ethnic character, civil conflicts or conflicts between countries, and how these shape women’s experiences. It would also be of importance to better understand the dynamics shaping attitudes and actions over SRHR for women taking an active role in conflicts (voluntarily or involuntarily) and how the dynamics in different armed groups influence women’s reproductive autonomy and decisions.

This review is not without limitations. The search was limited to peer-reviewed articles published in English between 2014 and 2024, which excluded relevant grey literature, non-English studies and older publications. This is particularly important in conflict-affected settings, where evidence is often produced by humanitarian organisations, local actors and programme implementers outside academic publishing. Furthermore, this review did not include women that had to flee to a foreign country due to war, therefore, it does not take into consideration the additional challenges that women may face as migrants or refugees. Also, since the included evidence was derived from women themselves, certain factors that are more relevant to healthcare professionals, aid workers or local governments/policymakers may have been overlooked. Finally, as no formal quality appraisal was conducted, the results should be interpreted as a mapping of available evidence rather than an assessment of the strength of that evidence.

## Conclusion

This scoping review shows that armed conflict shapes women’s family planning and abortion uptake and decision-making in complex and context-specific ways. Conflict can restrict access through insecurity, poverty, displacement, disrupted health systems and gender-based violence, while humanitarian responses may sometimes expand access to services. These findings highlight the need to examine the ways in which conflict-related changes in services, security, gender relations and social norms shape women’s reproductive health and autonomy.

## Supplementary Material

PRISMA_ScR_Checklist_11Sept2019.pdf

## Data Availability

All data supporting the findings of this scoping review are available within the article. No additional datasets were generated or analysed.

## References

[1] Starrs AM, Ezeh AC, Barker G, et al. Accelerate progress—sexual and reproductive health and rights for all: report of the Guttmacher–Lancet commission. Lancet. 2018;391:2642–15. doi: 10.1016/S0140-6736(18)30293-9 29753597

[2] United Nations Population Fund. Programme of action. adopted at the international conference on population and development, Cairo, 5-13 September 1994. New York: United Nations Population Fund; 2004.

[3] Canning D, Schultz TP. The economic consequences of reproductive health and family planning. Lancet. 2012;380:165–171. doi: 10.1016/s0140-6736(12)60827-7 22784535

[4] Hedstrom J, Herder T. Women’s sexual and reproductive health in war and conflict: are we seeing the full picture? Glob Health Action. 2023;16:2188689. doi: 10.1080/16549716.2023.2188689 36927249 PMC10026773

[5] Chi PC, Bulage P, Urdal H, et al. Perceptions of the effects of armed conflict on maternal and reproductive health services and outcomes in Burundi and northern Uganda: a qualitative study. BMC Int Health Hum Rights. 2015;15:7. doi: 10.1186/s12914-015-0045-z 25884930 PMC4392810

[6] Bendavid E, Boerma T, Akseer N, et al. The effects of armed conflict on the health of women and children. Lancet. 2021;397:522–532. doi: 10.1016/s0140-6736(21)00131-8 33503456 PMC7612212

[7] Amodu OC, Richter MS, Salami BO. A scoping review of the health of conflict-induced internally displaced women in Africa. Int J Environ Res Public Health. 2020;17:1280. doi: 10.3390/ijerph17041280 32079235 PMC7068277

[8] Pasquier E, Owolabi OO, Fetters T, et al. High severity of abortion complications in fragile and conflict-affected settings: a cross-sectional study in two referral hospitals in sub-Saharan Africa (AMoCo study). BMC Pregnancy Childbirth. 2023;23:143. doi: 10.1186/s12884-023-05427-6 36871004 PMC9985077

[9] Palmer JJ, Storeng KT. Building the nation’s body: the contested role of abortion and family planning in post-war South Sudan. Soc Sci Med. 2016;168:84–92. doi: 10.1016/j.socscimed.2016.09.011 27643843

[10] Aromataris E, Lockwood C, Porritt K, Pilla B, Jordan Z, editors. JBI manual for evidence synthesis: JBI. 2024 Available from: https://synthesismanual.jbi.global

[11] Tricco AC, Lillie E, Zarin W, et al. PRISMA extension for scoping reviews (PRISMA-ScR): checklist and explanation. Ann Intern Med. 2018;169:467–473. doi: 10.7326/M18-0850 30178033

[12] Munn Z, Barker T, Stern C, et al. Should I include studies from “predatory” journals in a systematic review? Interim guidance for systematic reviewers. JBI Evidence Synth. 2021;19:1915–1923. doi: 10.11124/JBIES-21-00138 34171895

[13] Norwegian register for scientific journals series and publishers. 2026. Available from: https://kanalregister.hkdir.no/en

[14] Lucas PJ, Baird J, Arai L, et al. Worked examples of alternative methods for the synthesis of qualitative and quantitative research in systematic reviews. BMC Med Res Methodol. 2007;7:4. doi: 10.1186/1471-2288-7-4 17224044 PMC1783856

[15] Namasivayam A, Arcos González P, Castro Delgado R, et al. The effect of armed conflict on the utilization of maternal health services in Uganda: a population-based study. PLoS Curr. 2017;9. doi: 10.1371/currents.dis.557b987d6519d8c7c96f2006ed3c271a PMC5693797 29188138

[16] Svallfors S, Billingsley S. Conflict and contraception in Colombia. Stud Fam Plann. 2019;50:87–112. doi: 10.1111/sifp.12087 30868587

[17] Torrisi O. Violent instability and modern contraception: evidence from Mali. World Dev. 2024;177:106538. doi: 10.1016/j.worlddev.2024.106538

[18] Ononokpono DN, Usoro NA, Akpabio EM. Non-use of modern contraceptives among women in humanitarian contexts: evidence from a qualitative study in Akwa Ibom State, Nigeria. J Biosoc Sci. 2023;55:199–212. doi: 10.1017/s0021932021000730 34986907

[19] Akseer N, Rizvi A, Bhatti Z, et al. Association of exposure to civil conflict with maternal resilience and maternal and child health and health system performance in Afghanistan. JAMA Netw Open. 2019;2:e1914819. doi: 10.1001/jamanetworkopen.2019.14819 31702799 PMC6902774

[20] Boddam-Whetham L, Gul X, Al-Kobati E, et al. Vouchers in fragile states: reducing barriers to long-acting reversible contraception in Yemen and Pakistan. Glob Health Sci Pract. 2016;4:94–108. doi: 10.9745/ghsp-d-15-00308 PMC4990166 27540129

[21] Curry DW, Rattan J, Huang S, et al. Delivering high-quality family planning services in crisis-affected settings II: results. Glob Health Sci Pract. 2015;3:25–33. doi: 10.9745/ghsp-d-14-00112 25745118 PMC4356273

[22] Akseer N, Wright J, Tasic H, et al. Women, children and adolescents in conflict countries: an assessment of inequalities in intervention coverage and survival. BMJ Glob Health. 2020;5:e002214. doi: 10.1136/bmjgh-2019-002214 PMC7042600 32133179

[23] Rivillas JC, Devia Rodriguez R, Song G, et al. How do we reach the girls and women who are the hardest to reach? Inequitable opportunities in reproductive and maternal health care services in armed conflict and forced displacement settings in Colombia. PLoS One. 2018;13:e0188654. doi: 10.1371/journal.pone.0188654 29346375 PMC5773007

[24] Kidman R, Palermo T, Bertrand J. Intimate partner violence, modern contraceptive use and conflict in the Democratic Republic of the Congo. Soc Sci Med. 2015;133:2–10. doi: 10.1016/j.socscimed.2015.03.034 25828259

[25] Casey SE, Gallagher MC, Kakesa J, et al. Contraceptive use among adolescent and young women in North and South Kivu, Democratic Republic of the Congo: a cross-sectional population-based survey. PLoS Med. 2020;17:e1003086. doi: 10.1371/journal.pmed.1003086 32231356 PMC7108687

[26] Erickson M, Goldenberg SM, Ajok M, et al. Structural determinants of dual contraceptive use among female sex workers in Gulu, northern Uganda. Int J Gynaecol Obstet. 2015;131:91–95. doi: 10.1016/j.ijgo.2015.04.029 26118326

[27] Casey SE, Cannon A, Mushagalusa Balikubirhi B, et al. Twelve-month contraceptive continuation among women initiating short- and long-acting reversible contraceptives in North Kivu, Democratic Republic of the Congo. PLoS One. 2017;12:e0182744. doi: 10.1371/journal.pone.0182744 28886016 PMC5590733

[28] Casey SE, Gallagher MC, Dumas EF, et al. Meeting the demand of women affected by ongoing crisis: increasing contraceptive prevalence in North and South Kivu, Democratic Republic of the Congo. PLoS One. 2019;14:e0219990. doi: 10.1371/journal.pone.0219990 31323055 PMC6641211

[29] Elmusharaf K, Byrne E, O’Donovan D. Social and traditional practices and their implications for family planning: a participatory ethnographic study in Renk, South Sudan. Reprod Health. 2017;14:10. doi: 10.1186/s12978-016-0273-2 28095917 PMC5240234

[30] Bove V, Di Salvatore J, Elia L, et al. Mothers at peace: international peacebuilding and post-conflict fertility. J Dev Econ. 2024;167:103226. doi: 10.1016/j.jdeveco.2023.103226

[31] Wirtz AL, Pham K, Glass N, et al. Gender-based violence in conflict and displacement: qualitative findings from displaced women in Colombia. Confl Health. 2014;8:10. doi: 10.1186/1752-1505-8-10 25076981 PMC4115473

[32] Bassel H. Acts of truth telling and testimony in the conceptualisation of reparations in post-conflict Peru. Global Soc. 2020;34:84–98. doi: 10.1080/13600826.2019.1668360

[33] Svallfors S, Båge K, Ekström AM, et al. Armed conflict, insecurity, and attitudes toward women’s and girls’ reproductive autonomy in Nigeria. Soc Sci Med. 2024;348:116777. doi: 10.1016/j.socscimed.2024.116777 38569280

[34] Svallfors S. Contraceptive choice as risk reduction? The relevance of local violence for women’s uptake of sterilization in Colombia. Popul Stud (Camb). 2022;76:407–426. doi: 10.1080/00324728.2021.1953118 34374637

[35] O’Brien ML. The consequences of the Tajikistani civil war for abortion and miscarriage. Popul Res Policy Rev. 2021;40:1061–1084. doi: 10.1007/s11113-020-09624-5 34658465 PMC8513771

[36] Erickson M, Goldenberg SM, Akello M, et al. Incarceration and exposure to internally displaced persons camps associated with reproductive rights abuses among sex workers in northern Uganda. J Fam Plann Reprod Health Care. 2017;43:201–209. doi: 10.1136/jfprhc-2016-101492 28183852

[37] Burkhardt G, Scott J, Onyango MA, et al. Sexual violence-related pregnancies in eastern Democratic Republic of Congo: a qualitative analysis of access to pregnancy termination services. Confl Health. 2016;10:30. doi: 10.1186/s13031-016-0097-2 28031743 PMC5175384

[38] Perera SM, Achakzai H, Giuffrida MM, et al. Barriers to seeking post-abortion care in Paktika Province, Afghanistan: a qualitative study of clients and community members. BMC Women’s Health. 2021;21:390. doi: 10.1186/s12905-021-01529-5 34742265 PMC8571834

[39] Asefa EY, Haile AB, Mohamed OY, et al. The magnitude of gender-based violence, health consequences, and associated factors among women living in post-war woredas of North Shewa zone, Amhara, Ethiopia, 2022. Front Glob Womens Health. 2024;5:1335254. doi: 10.3389/fgwh.2024.1335254 38774250 PMC11106405

[40] Koya MM, Mpinga EK. Perceptions of the rape crisis in the Eastern Democratic Republic of Congo: a community-based approach using an opportunistic design. Afr J Reprod Health. 2022;26:42–56. doi: 10.29063/ajrh2022/v26i4.5 37584983

[41] Tenaw LA, Aragie MW, Ayele AD, et al. Medical and psychological consequences of rape among survivors during armed conflicts in northeast Ethiopia. PLoS One. 2022;17:e0278859. doi: 10.1371/journal.pone.0278859 36508404 PMC9744300

[42] Oladeji O, Oladeji B, Chamla D, et al. Sexual violence-related pregnancy among internally displaced women in an internally displaced persons camp in Northeast Nigeria. J Interpers Violence. 2021;36:4758–4770. doi: 10.1177/0886260518792252 30095013

[43] Casey SE, Steven VJ, Deitch J, et al. “You must first save her life”: community perceptions towards induced abortion and post-abortion care in North and South Kivu, Democratic Republic of the Congo. Sex Reprod Health Matters. 2019;27:1571309. doi: 10.1080/09688080.2019.1571309 31533559 PMC7887765

[44] Casey SE, Isa GP, Isumbisho Mazambi E, et al. Community perceptions of the impact of war on unintended pregnancy and induced abortion in Protection of civilian sites in Juba, South Sudan. Glob Public Health. 2022;17:2176–2189. doi: 10.1080/17441692.2021.1959939 34323171

[45] Garry S, Checchi F. Armed conflict and public health: into the 21st century. J Public Health (Oxf). 2020;42:e287–e298. doi: 10.1093/pubmed/fdz095 31822891

[46] Svallfors S. Hidden casualties: the links between armed conflict and intimate partner violence in Colombia. Polit Gend. 2023;19:133–165. doi: 10.1017/S1743923X2100043X

[47] Johansson K, Sarwari M. Sexual violence and biased military interventions in civil conflict. Confl Manag Peace Sci. 2019;36:469–493. doi: 10.1177/0738894216689814

[48] Kvasnevska Y, Faustova M, Voronova K, et al. Impact of war-associated factors on spread of sexually transmitted infections: a systemic review [systematic review]. Front Public Health. 2024;12. doi: 10.3389/fpubh.2024.1366600 PMC11026856 38645454

